# Reconstruction of the three-dimensional beat pattern underlying swimming behaviors of sperm

**DOI:** 10.1140/epje/s10189-021-00076-z

**Published:** 2021-07-01

**Authors:** A. Gong, S. Rode, G. Gompper, U. B. Kaupp, J. Elgeti, B. M. Friedrich, L. Alvarez

**Affiliations:** 1 Center of Advanced European Studies and Research (caesar), Molecular Sensory Systems, Ludwig-Erhard-Allee 2, 53175 Bonn, Germany; 2 Theoretical Physics of Living Matter, Institute of Biological Information Processing and Institute for Advanced Simulation, Forschungszentrum Jülich, 52425 Jülich, Germany; 3 Biological Algorithms Group, TU Dresden, Cluster of Excellence ‘Physics of Life’ and Center for Advancing Electronics Dresden (cfaed), Helmholtzstr. 18, 01069 Dresden, Germany

## Abstract

**Abstract:**

The eukaryotic flagellum propels sperm cells and simultaneously detects physical and chemical cues that modulate the waveform of the flagellar beat. Most previous studies have characterized the flagellar beat and swimming trajectories in two space dimensions (2D) at a water/glass interface. Here, using refined holographic imaging methods, we report high-quality recordings of three-dimensional (3D) flagellar bending waves. As predicted by theory, we observed that an asymmetric and planar flagellar beat results in a circular swimming path, whereas a symmetric and non-planar flagellar beat results in a twisted-ribbon swimming path. During swimming in 3D, human sperm flagella exhibit torsion waves characterized by maxima at the low curvature regions of the flagellar wave. We suggest that these torsion waves are common in nature and that they are an intrinsic property of beating axonemes. We discuss how 3D beat patterns result in twisted-ribbon swimming paths. This study provides new insight into the axoneme dynamics, the 3D flagellar beat, and the resulting swimming behavior.

**Graphic abstract:**

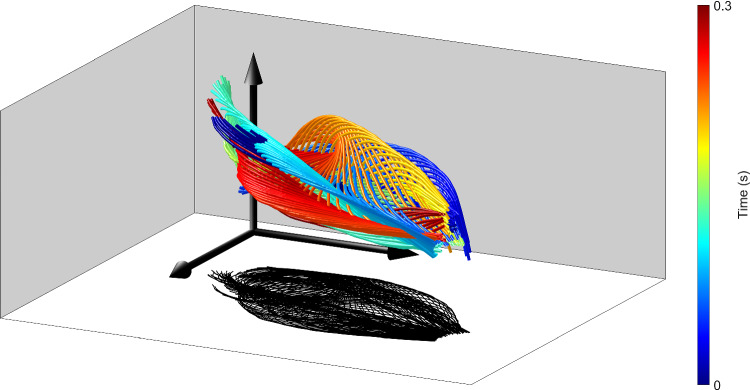

**Supplementary Information:**

The online version supplementary material available at 10.1140/epje/s10189-021-00076-z.

## Introduction

Sperm use the flagellum for sensing and self-propulsion to achieve fertilization [1–6]. Fertilization can occur externally or internally. Aquatic animals such as cnidarians, echinoderms, fishes, and amphibians [7] use external fertilization. Sperm and eggs are spawned into the water, i.e., eggs are fertilized outside the female body. Animals with external fertilization are referred to as external fertilizers. Fertilization in animals such as all mammals, birds, and reptiles occurs in the genital tract of the female body [8]. These animals are referred to as internal fertilizers.

Sperm cells from external and internal fertilizers encounter entirely different environments and may accordingly beat and swim differently to adapt to different challenges. Swimming has been mostly studied in sperm confined to the glass-water interface (2D) because conventional microscopy such as bright-field, dark-field, or epifluorescence microscopy can only image a plane. Consequently, these techniques only provide 2D or semiquantitative 3D information [9, 10].

**Fig. 1:**
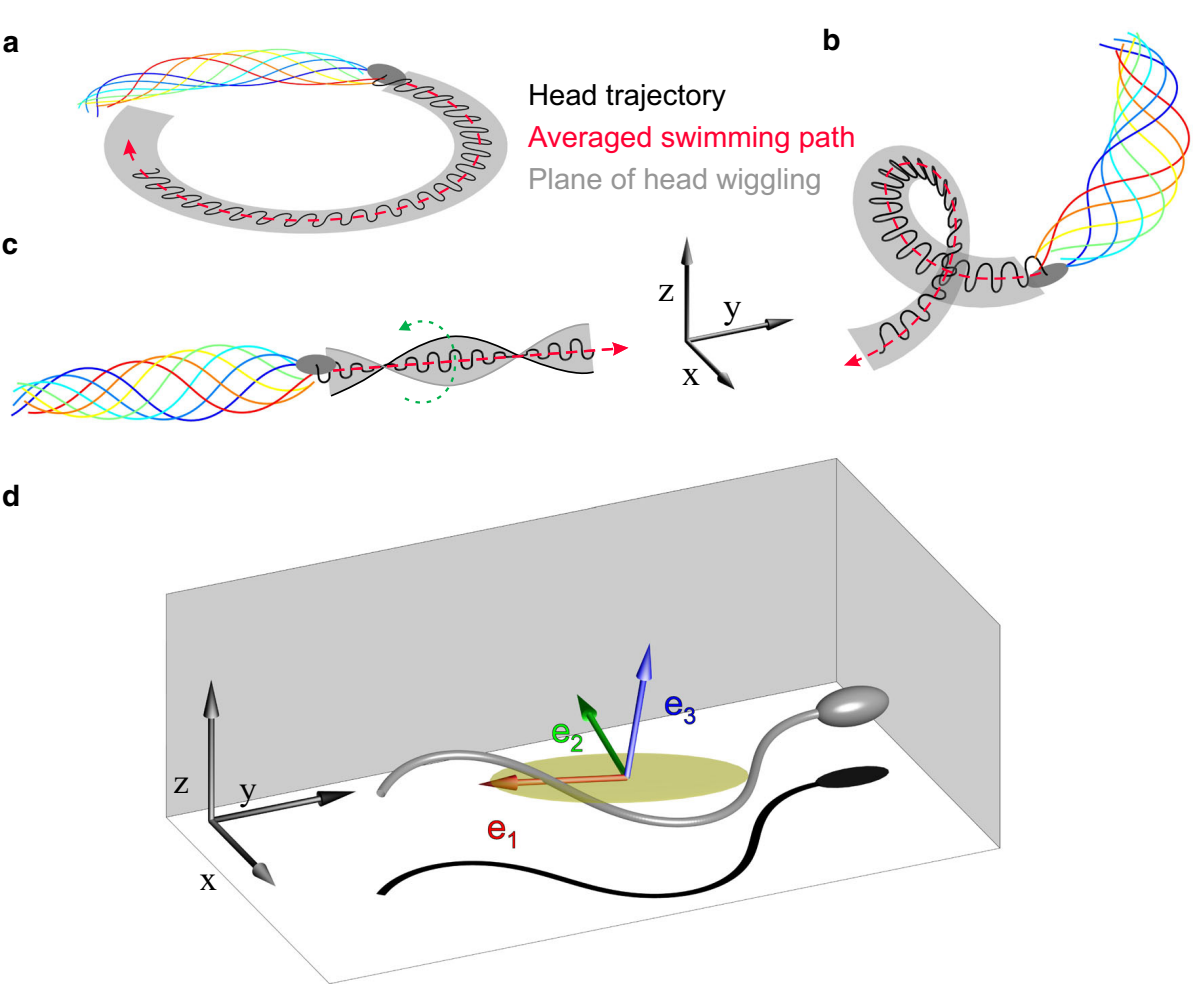
Stereotypical swimming trajectories and averaged paths of sperm. **a**–**c** Sperm cells are propelled in a liquid by regular bending waves of their slender flagellum (sequence of flagellar shapes shown in rainbow colors). The trajectory of the sperm head (black line) is characterized by a wiggling motion at the frequency of the flagellar beat. The net motion of the sperm cell is described by the averaged path (red dashed arrow), obtained by averaging out the rapid head wiggling, as well as the surface described by the moving local plane of head wiggling (gray surface). **a** Circular averaged path and corresponding head trajectory confined to an annulus region. **b** Helical averaged path and corresponding helical ribbon characterizing the wiggling head trajectory. The helical averaged path is left-handed. **c** Curvilinear averaged path and corresponding twisted ribbon characterizing the wiggling head trajectory. The beat plane rotates clockwise when viewed from the head towards the distal end of the flagellum. This corresponds to counterclockwise rotation with respect to the $\mathbf{e}_1$ vector (introduced below), which approximately points from the proximal to distal direction, and hence a positive rolling rate $\Omega _{\mathrm{roll}}>0$ in our convention. The green arrow indicates the rotation direction of the beat plane. Black arrows represent the laboratory reference frame. The trajectories were computed using resistive-force theory from prescribed beat patterns: an asymmetric planar flagellar beat (**a**), an asymmetric non-planar flagellar beat (**b**), and a symmetric non-planar flagellar beat (**c**) (adapted from [21]). **d** Reference frame defined by the gyration tensor of the cell. The gyration tensor describes the second moments for each flagellar shape. The eigenvectors $\mathbf{e}_1$, $\mathbf{e}_2$, and $\mathbf{e}_3$ of this tensor define a reference frame that translates and rotates with the sperm cell. This co-moving reference frame (colored arrows) is shown in the laboratory *xyz*-reference frame (black arrows). The square root of the eigenvalues of the gyration tensor can be used as a measure of the flagellar extension along the direction of each eigenvector. An ellipsoid (yellow) with semi-axes equal to the square root of the eigenvalues of the flagellar shape is shown

While swimming, the sperm head wiggles at the frequency of the flagellar beat due to counterbalancing forces of the actively beating flagellum. We will refer to this wiggling trajectory of the center of the sperm head as *head trajectory*. Averaging out the fast head wiggling yields the *averaged path* (Fig. 1).

Close to boundary surfaces, sperm from external fertilizers such as sea urchins [11], starfish [12], and freshwater fish [13], to list a few, swim along an averaged path describing a circle (Fig. 1a). Analysis of the 2D projection of the flagellum shows that, while swimming in circles, the flagellar beat pattern is asymmetric. Moreover, most of the flagellum is in focus, suggesting that the beat pattern is almost, albeit not entirely, planar [14]. In internal fertilizers such as mammals, the most prominent averaged paths are circles or curvilinear paths, which might appear as straight lines [15, 16]. Circular averaged paths are caused by an asymmetric flagellar beat [15], whereas straighter averaged paths are caused by a more symmetric beat.

Using holography to track the sperm head in three space dimensions (3D) has advanced our understanding of 3D swimming for sea urchin, the model system of sperm chemotaxis research, and also mammalian sperm [17–20]. Far from boundary surfaces, sea urchin sperm swim on helical averaged paths, and the wiggling head moves within the surface of a helical ribbon (Fig. 1b). The curvature and torsion of the helical path are precisely tuned for an intriguing helical klinotaxis strategy that facilitates deterministic chemotactic navigation in a chemical gradient [21]. Sperm from horse and human mostly swim on 3D curvilinear paths, and the wiggling head moves within the surface of a twisted ribbon (Fig. 1c). A smaller fraction of horse and human sperm swim on helical or planar averaged paths [17, 18].

Going beyond tracking of the sperm head and determining the 3D flagellar beat that controls the different swimming behaviors is challenging. In fact, only a limited number of studies have recorded the 3D flagellar beat or reconstructed it from 2D data [10, 20, 22, 23]. Machemer manually tracked stereographic 2D projections of beating cilia anchored on the epithelial surface of a swimming *Paramecium* cell [24]. Wilson et al. pioneered digital inline holographic microscopy to study 3D swimming of flagellated Malaria parasites [23]. Recently, Gadelha et al. harnessed a rapidly oscillating microscope objective driven by a piezoelectric device to track 3D beat patterns of sperm Ref. [25]. However, we are just beginning to understand 3D beat patterns. In particular, the link between the beat pattern and the underlying swimming trajectories is still ill-defined.

To characterize flagellar beat patterns requires appropriate reference frames. The flagellar beat will be periodic only with respect to a frame that translates and rotates together with the cell, i.e., a co-moving frame. Previous studies used a head-fixed frame [15, 25, 26] or the principal axes of the gyration tensor of the flagellar shape as a natural choice of a co-moving frame [10]. In the following, we will employ a co-moving frame derived from the gyration tensor to describe the flagellar beat and a stationary laboratory *xyz*-reference frame to describe the swimming paths of sperm (Fig. 1d).

Using digital inline holographic microscopy (DIHM), we report the 3D flagellar beat pattern underlying swimming behaviors of invertebrate (sea urchin) and mammalian (human) sperm near a boundary surface. We show that sea urchin sperm feature an almost planar beat pattern. In contrast, mammalian sperm display a pronounced non-planar beat pattern characterized by torsion waves that peak at those flagellar portions where the curvature is low. We speculate that such torsion waves may be a ubiquitous feature of flagellar bending waves and may inform models of motor control underlying flagellar bending waves.

## Results

**Fig. 2:**
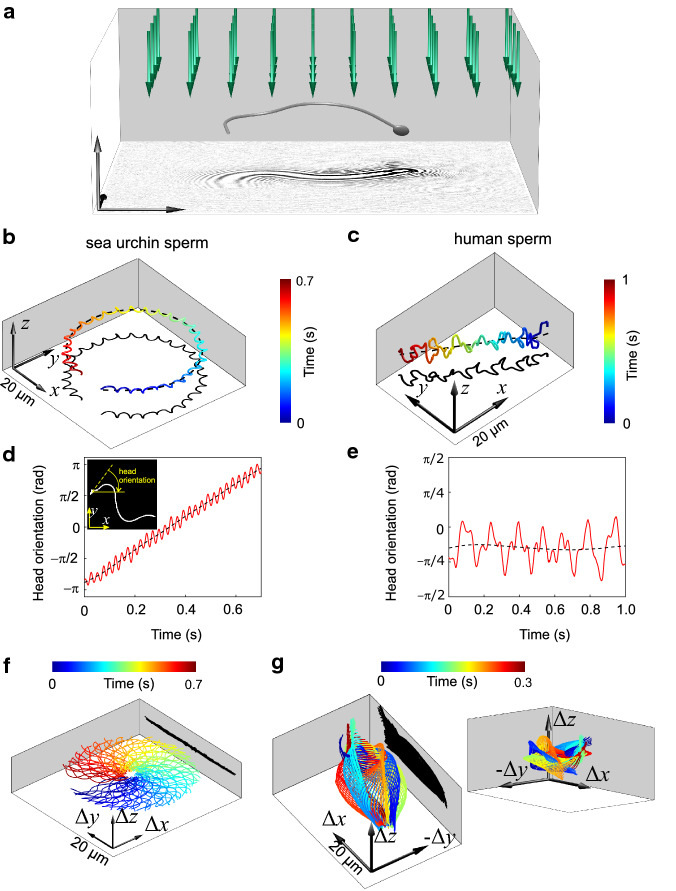
Holographic reconstruction of the swimming trajectories, head orientation, and 3D flagellar shape of sea urchin and human sperm swimming near a boundary surface. **a** Schematic of a holographic recording. Coherent laser light (510 nm in this study) is used to illuminate the sperm cell (indicated by green arrows). Transmitted and scattered light generate an interference pattern (hologram shown at the bottom) that is imaged by the microscope onto the camera detector. The hologram can be used to reconstruct the sperm cell in 3D. **b** Reconstructed head trajectory of a sea urchin sperm cell. The averaged path is a circle, and the head trajectory is confined to an annulus. **c** The reconstructed head trajectory of a human sperm cell. The averaged path is curvilinear, and the head trajectory describes a twisted ribbon. The color code represents time. The black curve is the 2D projection of the trajectory. The dashed black line is the averaged path. **d**–**e** Angle of head orientation of sea urchin (**d**) and human (**e**) sperm (obtained from a projection on the laboratory *xy*-plane). The black dashed line denotes a time-averaged head-orientation angle. Inset: definition of the head-orientation angle from a projection on the laboratory *xy*-plane. The white color indicates the projection of the sea urchin sperm. The yellow dashed line is aligned with the long axis of the sperm head. The horizontal solid yellow line indicates the positive direction of the *x*-axis. For sea urchin, the head-orientation angle oscillates around a slowly increasing time-average, reflecting an averaged path that is circular (**d**). In contrast, for human sperm, the head- orientation angle oscillates around an approximately constant value, reflecting an averaged path that is almost straight (**e**). **f**, **g** Sequence of three-dimensional flagellar shapes in the stationary laboratory *xyz*-frame where the *xy*-position of the head center has been aligned. **f** The flagellar-beat envelope of the sea urchin sperm is almost flat, which can be better visualized by the side projection (in black) on the side wall. See movie 1. **g** In contrast, the flagellar-beat envelope of human sperm features a larger variation in the *z*-direction. For human sperm, two different camera views are shown. See also movie 2

The relationship between flagellar beat pattern and head trajectory has been studied numerically using resistive-force theory [15, 21, 27]. Assuming a periodic beat pattern as input, theory predicts three types of averaged paths - circular, helical, and curvilinear (Fig. 1); the corresponding envelope of the wiggling head trajectory represents either an annulus, a helical ribbon, or a twisted ribbon, respectively. Two key features of the flagellar beat (corresponding to broken symmetries) determine the type of trajectory: in-plane asymmetry of flagellar bending waves and an out-of-plane component. Using DIHM (Fig. 2a), we tested these predictions experimentally by recording the head trajectories of sperm from sea urchins and humans near the boundary surface of an observation chamber. We consider a stationary laboratory frame with coordinate *x*- and *y*-axis parallel to the boundary surface and *z*-axis pointing away from the center of the planet earth (antiparallel to the optical axis of the microscope) (Figs. 1d and 2a). Sea urchin sperm swam along a circular averaged path (Fig. 2b). The swimming parameters were: speed along the averaged path $v_{\mathrm{ap}}= 191 \pm 29\,\upmu \hbox {m/s}$, circle radius $r = 31.2\,\pm 3.7\,\upmu \hbox {m}$ (track duration 2 s; $n = 10$ cells, mean ± s.d.). The head trajectory displayed the expected characteristic head wiggling; the envelope represents an almost planar annulus that is coplanar with the *xy*-plane (compare Figs. 1a and 2b). The maximal deviation in the z-direction (normal to this annulus) was $\Delta z = 0.78 \pm \,0.19\, \upmu \hbox {m}$. Head wiggling was also apparent from a plot of the head-orientation angle vs. time (Fig. 2d). Using head wiggling as a proxy for the beat frequency, we estimated a beat frequency of $38.4 \pm 3.7\hbox { Hz}$ ($n = 10$). In addition to the fast oscillation reflecting head wiggling, the head-orientation angle increased with an approximately constant rate, consistent with swimming along a circular path at a constant speed. This rate provides the rotation velocity of yawing (rotation around the *z*-axis), $\Omega _{\mathrm{yaw}} = 6.2 \pm \, 1.2\hbox { rad/s}$ ($n = 10$). We note the geometric relation $\Omega _{\mathrm {yaw}}\approx$ $v_{\mathrm {ap}}/\textit{r,}$ which relates the yaw rotational velocity, swimming speed $v_{\mathrm {ap,}}$ and radius *r* of the swimming circle.

The averaged path of human sperm was straight on a time scale of a few seconds. The head trajectory displayed a pronounced 3D component compared to sea urchin sperm (Fig. 2c). As the sperm cell moved forward, the plane of lateral head wiggling slowly rotated around the averaged path. This movement is consistent with a flagellar beat that is approximately planar. On a short timescale, this results in head wiggling within a plane. Yet, on longer timescales, the out-of-plane component of the flagellar beat causes a rotation of this plane of head wiggling. This peculiar type of swimming path is known as *twisted ribbon* [18, 21]. The swimming parameters were: $v_{\mathrm {ap}}= 49 \pm 19\,\upmu \hbox {m/s}$, maximum height change in the *xyz*-laboratory reference frame $\Delta z = 6.7 \pm 3.2\, \upmu \hbox {m}$, and $\Omega _{\mathrm {yaw}} = 0.0 \pm 0.2\hbox { rad/s}$ (track duration 2 s; $n = 12$). The average head-orientation angle was almost constant for human sperm (Fig. 2e), consistent with their straight averaged path.

Any 3D beat pattern–and, in fact, any space curve that varies in time - can be fully characterized by its curvature profile $$\upkappa (s$$, *t*) and torsion profile $$\uptau (s$$, *t*) of the centerline of the flagellum as a function of arc-length *s* and time *t*. A beat pattern with zero torsion, $$\uptau (s$$, $$t) = 0$$, is planar and results in a planar circular swimming path. If the curvature of the beat pattern after half a beat cycle equals its mirror image, $$\upkappa (s,t) = -\upkappa (s,\,t + T/2$$), the swimming circle degenerates into a straight line. In general, a 3D beat pattern will result in a helical swimming path. However, the helical path can become a straight path when the radius of the helix approaches zero. For example, Fig. 1c displays the straight swimming path resulting from a symmetric sinusoidal beat with constant torsion. The head-wiggling plane forms a twisted ribbon.

We analyzed the 3D flagellar beat of sea urchin and human sperm to test these predictions. The 3D beat pattern of flagella relative to the *xyz*-laboratory reference frame is shown in Fig. 2f, g, and movies 1 and 2 with the respective head position aligned (i.e., flagellar shapes were translated but not rotated). The superposition of flagellar waveforms is almost flat for sea urchin sperm (Fig. 2f), whereas the envelope of the flagellar beat of human sperm appears like a twisted star fruit with four ridges (Fig. 2g). The beat pattern of human sperm displayed a larger out-of-plane component ($\Delta z \approx 7\,\upmu \hbox {m}$) compared to that of sea urchin sperm ($\Delta z \approx 1\,\upmu \hbox {m}$). We conclude that sea urchin sperm have an almost planar beat. By contrast, for human sperm, the large change in the *z*-coordinate requires further investigation, as it may indicate either a non-planar beat or, possibly, a planar beat whose plane of beating is tilted with respect to the *xy*-plane.

**Fig. 3:**
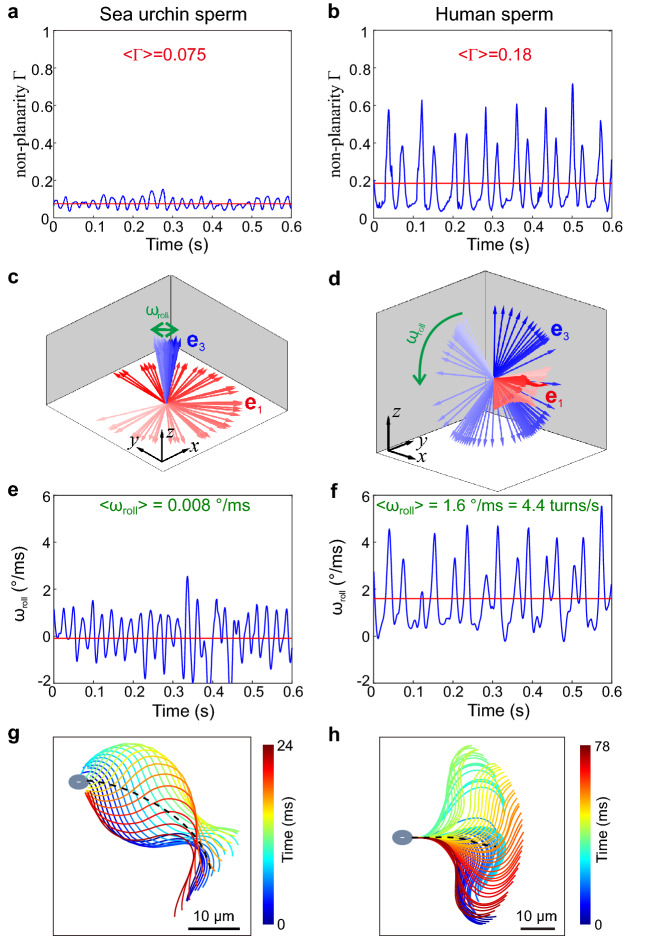
The gyration tensor characterizes the beat pattern of sea urchin and human sperm and allows visualization of sperm rolling. **a**–**b** Non-planarity ratio $\Gamma$ of sea urchin sperm (**a**) and human sperm (**b**) defined in terms of the gyration tensor. The non-planarity ratio of human sperm is much greater compared to that of sea urchin sperm. **c**–**d** Elongation vector $\mathbf{e_1}$, (red) and vector normal to the beat plane $\mathbf{e_3}$ (blue) for sea urchin sperm (**c**) and human sperm (**d**); see main text for their definitions as principal axes of the gyration tensor. The hue of the arrows represents time. Vectors are drawn relative to the stationary laboratory reference frame. The green arrow indicates the rotation of the $\mathbf{e_3}$ vector. For sea urchin, the $\mathbf{e_3}$ vector is always pointing in approximately the same direction, consistent with swimming along a planar circular averaged path. For human sperm, the $\mathbf{e_3}$ vector continuously rotates, consistent with cell rolling. (**e**-**f**), Rotational velocity of the normal vector $\mathbf{e_3}$ of the beat plane for sea urchin sperm (**e**) and human sperm (**f**). The rotational velocity of $\mathbf{e_3}$ cell oscillates around zero for sea urchin sperm, whereas for human it is always larger than zero and oscillates with time, indicating that the cell is continuously rolling (as opposed to oscillatory wobbling). (**g**–**h**), Superposition of consecutive flagellar shapes of sea urchin (**g**) and human sperm (**h**) in the plane described by the vectors $\mathbf{e_1}$ and $\mathbf{e_2}$ of the co-moving reference frame. The cells have been additionally translated and rotated in this plane to align their head position and make the long axis of the head parallel to the horizontal axis. The flagellum of sea urchin sperm beats asymmetrically, being inclined toward one side of the head’s long axis. The time average over one beat period is shown as a dashed line and represents a measure of the mean flagellar curvature. The projection of the flagellar beat of human sperm shows a symmetric pattern, and the time average is more parallel to the long axis of the sperm head compared to that of sea urchin sperm, indicating a low mean flagellar curvature. Averages for exemplary sperm cells shown. Averages across cells are presented in the text

To distinguish between these possibilities, we used a gyration-tensor analysis (see [10] and Fig. 1d). The gyration tensor **G** quantifies the second moments of the flagellar shape at a given time point (similar to the moment-of-inertia tensor). By diagonalizing the gyration tensor **G**, we obtain three orthogonal axis-vectors $(\mathbf{e} _{{1}}$, $\mathbf{e} _{{2}}$, $\mathbf{e} _{{3}})$ and their corresponding eigenvalues ($\lambda _{{1}}$, $\lambda _{{2}}$, $\lambda _{{3}}$). These eigenvectors represent the principal axes, and their corresponding eigenvalues represent the variation of the flagellar shape along each axis (Fig. 1d). We assume that the principal vectors are ordered by the value of their corresponding eigenvalues, $\lambda _{{1}}\,\ge \lambda _{{2}} \ge \lambda _{{3}}$. The vector $\mathbf{e} _{{1}}$ is the principal vector corresponding to the direction along which the projection of the flagellar shape is maximal and can serve as a proxy for the long axis of the flagellum; $\mathbf{e} _{{2}}$ marks the direction along which deviations of the flagellar shape from a straight line are maximal. This coarsely corresponds to the beat direction that together with $\mathbf{e} _{{1}}$ defines the instantaneous plane of flagellar beating; finally, $\mathbf{e} _{{3}}$ marks the direction normal to this beat plane. The square root of the eigenvalues has units of a length and can be interpreted as “axis lengths” of the flagellum for the respective direction: $r_{1} =\sqrt{\lambda _{1} }$ is the half-elongation length, $r_{2} =\sqrt{\lambda _{2} }$ characterizes the amplitude of the flagellar bending wave, and $r_{3} =\sqrt{\lambda _{3} }$ characterizes the out-of-plane beat component. The *non-planarity ratio* ($\Gamma$), a measure of planarity of a flagellar shape, is defined as $r_{{3}}/r_{{2}}$. If $\Gamma$ is close to zero, the flagellar shape is almost planar. Example time series of the non-planarity ratio for sea urchin and human sperm are shown in Fig. 3a and b. For sea urchin sperm, the time average of the non-planarity ratio is small ($\langle \Gamma \rangle = 0.09 \pm 0.02, n =11$ cells). For human sperm, the non-planarity ratio oscillates (between 0.1 to 0.6), and its time average is considerably larger ($\langle \Gamma \rangle = 0.28 \pm 0.10, n = 17$ cells) compared to sea urchin sperm.

Previous studies have exploited changes in the 2D projection of the sperm head as a function of time as a proxy for the orientation of the sperm head in 3D space [28–30]. Whether the observed changes are due to the rolling of the sperm cell around its long axis or rather wobbling of the flagellar beat plane is controversial [30–32]. Rolling refers to a continuous rotation of the flagellar beat plane around the local tangent of the averaged swimming path. By contrast, wobbling refers to an oscillatory rolling motion of the beat plane in one direction followed by rolling into the opposite direction. The 3D tracking of flagellar beat patterns allowed discriminating between rolling and wobbling by inspecting the orientation of the vector normal to the beat plane $\mathbf{e} _{{3}}$ (Fig. 3c and d). For sea urchin sperm, the $\mathbf{e} _{{3}}$ vector, although slightly wobbling, is approximately pointing in the same direction. Concomitantly, the $\mathbf{e} _{{1}}$ vector is rotating continuously, consistent with a circular swimming path of sea urchin sperm. For human sperm, the $\mathbf{e} _{{1}}$ vector is always pointing approximately in the same direction, while the $\mathbf{e} _{{3}}$ vector rotates continuously. We can define a rotational velocity of $\mathbf{e} _{{3}}$ for rotations around $\mathbf{e} _{{1.}}$ For sea urchin sperm, this rotational velocity oscillates with a small amplitude around zero (Fig. 3e). In contrast, for human sperm, the rotation velocity of $\mathbf{e} _{{3}}$ is always positive (Fig. 3f). This shows that human sperm invariably roll in one direction consistent with Schiffer and coworker’s result [32]. The mean rolling speed for human sperm was $7.8 \pm 2.7$ turns/s ($n = 14$ cells). Irrespective of whether sperm swim at the upper or lower surface of the observation chamber, all sperm rolled clockwise when viewed from the head towards the flagellum’s distal tip (eight cells near the lower surface and ten near the upper surface have been analyzed; Fig. 1c). This result is consistent with a previous study [10]. It is noteworthy, when sperm roll, a simple 2D projection of their flagellum will contain frequency contributions due to rolling, and the projected flagellar beat may appear not periodic anymore.

To quantify the asymmetry of flagellar bending waves in the (rotating) plane of flagellar beating, we need to switch to a co-moving coordinate frame that rotates together with the sperm cell. Therefore, we projected flagellar shapes on the plane spanned by the principal vectors $\mathbf{e} _{{1}}$ and $\mathbf{e} _{{2}}$ of the gyration tensor with the origin at the center of the head. This was followed by an in-plane rotation of the cell to align the long head axis with the horizontal axis (intuitively, we thereby ‘undo’ the rolling of swimming sperm).

For sea urchin sperm, this projected flagellar beat is highly asymmetric (Fig. 3g). The time-averaged shape of the flagellum forms a curved arc (curvature is $31 \pm 3.7\hbox { mm}^{{-1}}$, $n = 6$ cells, tracking duration 2 s). By contrast, the projection of the beat of human sperm displayed a much more symmetric shape (Fig. 3h). The curvature of time-averaged flagellar projection was $7.9 \pm 6.5\hbox { mm}^{{-1}}\,(n = 8$ cells, tracking duration 2 s).

**Fig. 4:**
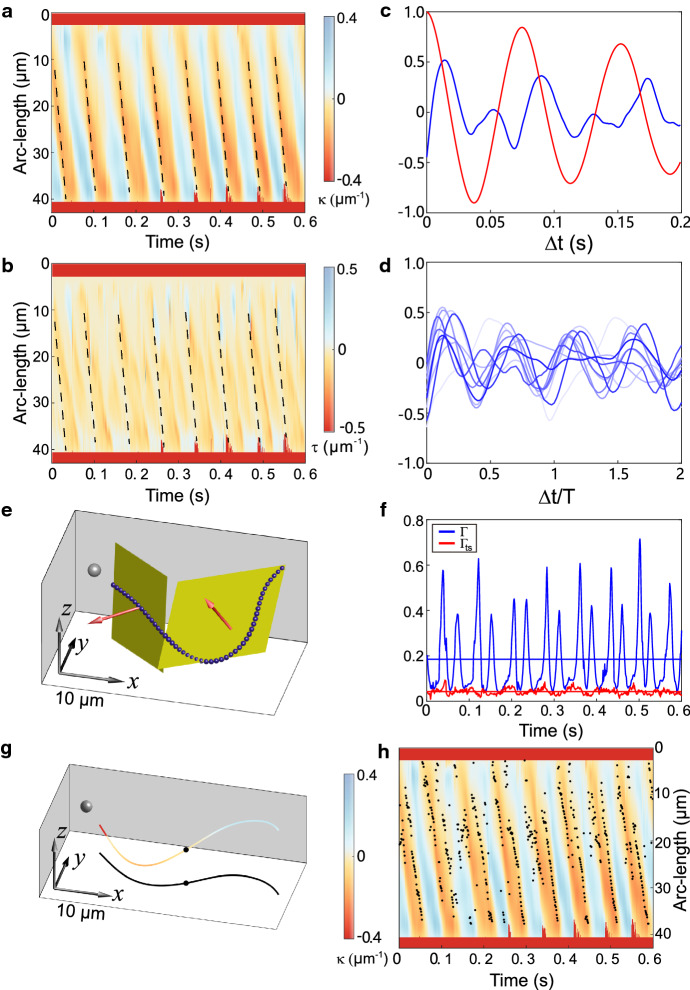
Illustration of flagellar shape, curvature ($\upkappa$), and torsion ($\uptau$) of human sperm. **a**–**b** Kymographs of flagellar curvature (**a**) and torsion (**b**). Both flagellar curvature and torsion show a traveling wave pattern. Black dashed lines indicate the wavefront propagation. **c** Normalized autocorrelation of the curvature (red) and cross-correlation between the absolute value of curvature and torsion (blue) at arc-length position $30\,\upmu \hbox {m}$ (from the proximal end) as function of time. The cross-correlation reveals that torsion peaks are phase-shifted by roughly one-quarter of a beat period. **d** Cross-correlation between curvature and torsion for $n = 10$ sperm cells. Time has been normalized to the beat period *T* measured from the curvature’s autocorrelation function. **e** The flagellum of human sperm can be segmented into two almost flat segments. The yellow panes indicate the respective osculating planes of the two flat segments with respective normal vectors (red), which are connected at the torsion point. The gray sphere represents the center of the sperm head. **f** Non-planarity ratio $\Gamma$ (red) and the two-segment non-planarity $\Gamma _{\mathrm{ts}}$ (blue) time series for the same human sperm cell. The horizontal lines indicate the respective mean of these curves. Of note, $\Gamma _{\mathrm{ts}}$ is significantly smaller than $\Gamma$. **g** The flagellar curvature color-coded along the human flagellum to illustrate its relationship with the torsion point (black circle). The black line represents the projection of the flagellum on the *xy*-plane. **h** Same as panel a, with super-imposed torsion points (black circles): torsion points are predominantly located near the zero-curvature region. Dark red in (**a**), (**b**), (**g**), and (**h**) represent missing curvature and torsion values

The time-dependent curvature of flagellar shapes (see Methods section for the definition and sign convention) and torsion for human sperm reveal a traveling wave pattern (Fig. 4a and b and Fig. S1). Torsion was predominantly negative, i.e., flagellar shapes are homochiral with defined handedness (Fig. 4b). Torsion and curvature waves displayed a phase shift of roughly one-quarter of a beat period (Fig. 4c–d). Thus, the torsion peaked in the straighter flagellar regions where the curvature is low. The zero-crossing of the signed curvature marks an inflection point of the flagellar shape where it transits from a concave to a convex shape. For human sperm swimming in low viscosity media, the wavelength of the traveling curvature wave is comparable to the length of the flagellum [33]; correspondingly, we observe a single low-curvature region along the flagellum.

As torsion can be challenging to compute from noisy data, we performed a second, more robust analysis to quantify 3D beat patterns: the measured curvature and torsion profiles of human sperm flagella suggest that flagellar shapes can be approximated as a concatenation of two planar segments that are twisted relative to each other (Fig. 4e).

To further characterize this putative flagellar shape, we propose a parameter, called here *two-segment non-planarity* ($\Gamma _{\mathrm {ts}})$. This parameter is defined as a weighted mean of the respective non-planarity ratios of two flagellar segments, obtained by splitting the flagellar shape at arc-length position *s*. Specifically, we first define the following function, where the splitting point *s* is still a free parameter

1 $$\begin{aligned} {{\bar{\Gamma }}}\text{( }s\text{) }=\frac{s\Gamma _{1} }{L}+\frac{(L-s)\Gamma _{2} }{L}. \end{aligned}$$

Here, *s* represents the arc-length position of the split point along the flagellum, *L* is total arc-length, and $\Gamma _{{1}}$, $\Gamma _{{2}}$ are the non-planarity scores for the first and the second segment, respectively. The two-segment non-planarity is now defined as the minimum

2 $$\begin{aligned} \Gamma _{\mathrm{ts}} =\min [{{\bar{\Gamma }}}\text{( }s\text{)] }. \end{aligned}$$

We call the location *s* where ${\bar{\Gamma }}\text{( }s\text{) }$ becomes minimal the *torsion point*. Figure 4f shows a comparison between $\Gamma$ and $\Gamma _{\mathrm {ts}}$ for a human sperm cell. The $\Gamma _{\mathrm {ts}}$ is dramatically smaller when compared to $\Gamma$ (Fig. 4f). This suggests that flagellar shapes for human sperm are approximately composed of two nearly planar segments. Such a twisted-plane shape has previously been proposed for the flagellar shape of hamster sperm [34]. The transition point $s_{\mathrm {ts}} =\hbox { argmin}[{\bar{\Gamma }}(s)]$ between the two planar segments marks the position of a torsion point between the two planes and is positioned at the low-curvature flagellar segments (Fig. 4g and h). This result is consistent with the presence of torsion peaks located at low-curvature regions obtained by the first method that seeked to estimate torsion directly. We conclude that the low-curvature region (straight part) splits the flagellum into two almost planar curves.

**Fig. 5:**
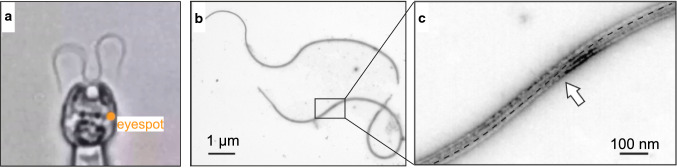
**Illustration of the out-of-plane component and the central pair (CP) twist in flagella of** *Chlamydomonas reinhardtii*. **a** Image of the green alga *C*. *reinhardtii*. The cell is held with a micropipette (bottom). While parts of both flagella are in focus, the blurred middle part of the left flagellum indicates that this portion lies out of the focal plane. The transitions occur near the straight regions of the flagellum. Figure adapted from [54]. **b** Electron-microscopy image of two isolated flagella from *C*. *reinhardtii*. **c** Zoom-in of the box in (**b**). The dashed line marks the C1 microtubule in the CP. The arrow points to the torsion point. Panels (**b**) and (**c**) are adapted from [55]

## Discussion

We identified characteristic differences in the trajectory and three-dimensional flagellar waveform of sea urchin and human sperm swimming near a boundary surface. The flagellar beat of sea urchin sperm is almost planar with a pronounced asymmetry in the beat plane: this results in a circular averaged swimming path. Concomitantly, the wiggling trajectory of the sperm head stays within a planar annulus. The beat plane stays approximately parallel to the boundary surface and exhibits only weak oscillatory wobbling of small amplitude. By contrast, the flagellar beat of human sperm is non-planar, with a projected shape that is roughly symmetric: this results in curvilinear averaged paths. Due to rolling of the entire cell, caused by the pronounced out-of-plane component of the beat pattern, the plane of head wiggling continuously rotates and describes a twisted ribbon. For both sea urchin and human sperm, the observed swimming patterns are consistent with theoretical predictions [21]. It is noteworthy that sea urchin sperm, while swimming far from boundary surfaces, move on a helical averaged path in 3D [21, 35]. Previous theory highlighted that a small out-of-plane component would be sufficient to account for helical swimming paths [21]. We attempted to measure flagellar beat patterns of sea urchin sperm swimming far from boundary surfaces along helical averaged paths, yet did not succeed with the present experimental setup.

Due to propulsion, steric, and hydrodynamic interactions, sperm naturally accumulate at boundaries (see [36] and references therein). The observation that sea urchin sperm swim along planar circles near a boundary surface could be explained by hydrodynamic interactions with the boundary surface, which restricts the flagellum to beat in a plane [37] with corresponding planar circular swimming path. Yet, we observe that these interactions do not result in planar swimming of human sperm cells. Previous theoretical studies [38] have shown that computer models of sperm featuring a non-planar flagellar beat can swim in tight circles near a boundary wall with no rotation of the beat plane provided that the mean flagellar curvature is high. By contrast, sperm whose flagellum features a low mean curvature are predicted to swim along curvilinear paths while their beat plane rotates. These theoretical predictions are consistent with the measured swimming paths and flagellar beat patterns reported here for sea urchin and human sperm.

A planar beat may cause enhanced hydrodynamic attraction to the boundary surface, as the sperm cell can stay very close to the surface [38]. However, for a curved surface, like in curved micro-channels or the surface of an egg, a non-planar beat might be advantageous for keeping sperm close to the surface, as the enhanced repulsion of the tail from the wall generates an effective sperm tilt, which together with propulsion keeps the sperm head close to the surface [39].

Our data strongly suggests that human sperm cells display traveling torsion waves with traveling torsion peaks that co-localize with the region of low curvature (i.e., the straight region) of flagellar shapes. For sea urchin sperm, we could not reliably quantify the flagellar torsion because their flagellar beat is almost planar.

How general is this finding of traveling torsion waves? In the following, we argue that torsion waves of the 3D beat may be more general in nature. For example, a careful inspection of the beating flagella from the green algae *Chlamydomonas reinhardtii* (Fig. 5a) reveals that at the selected time point, the middle part of the left flagellum is blurred, indicating that this part is not in the same focal plane as the most proximal and most distal parts. Intriguingly, the transition between blurred and in-focus flagellar parts occurs in a region where the flagellum is approximately straight. Although no quantitative analysis is available, it is plausible that this flagellum has a twisted-plane shape, similar to the one described here for human sperm. Alternatively, the flagellar beat could be approximately planar with a beating plane tilted with respect to the imaging plane. A non-planar beat has been suggested in recent work [40]. Chiral flagella beating in *Chlamydomonas* is important for helical swimming and phototaxis. Future quantification with an increased resolution of the 3D beat of *Chlamydomonas* is required to discern between alternative beat shapes.

Two lines of evidence shed light on the origin of the torsion point. First, a recent cryo-EM study shows that most dynein motors are active in the straight region [41]. Dyneins are arranged in inner and outer dynein arms that invariably project from sub-tubules A to sub-tubules B of the flagellar axoneme. When all motors in the flagellar circumference are active, the forces exerted by all dyneins are counterbalanced at this region, and, consequently, this flagellar region is not bent. However, due to the asymmetric projection of dyneins from A to B sub-tubules, the flagellum is presumably twisted in the straight flagellar regions [42]. Second, the central pair (CP) of the *C. reinhardtii* flagellum can rotate and has a preferred orientation: the C1 central microtubule always stays at the convex side of the flagellum (Fig. 5b and c). Thus, the CP is twisted at the inflection point, i.e., the straight region between bends.

The CP might tune the mechanical properties of different axonemes, contributing to an anisotropic bending rigidity. The bending and twist rigidity are key determinants of emergent flagellar bending waves. Computer models of sperm predict that even for flagellar bending waves generated by a planar force pattern, the drag of the flagellum in the fluid might result in a non-planar beat. This elastic instability occurs when the twist rigidity is sufficiently low compared to the bending rigidities [39, 43]. Thus, conditions that increase hydrodynamic forces such as increasing the beat amplitude, wavelength, or high media viscosity are expected to enhance buckling, which would result in a larger out-of-plane component under these conditions. The CP could play a role in tuning the twist rigidity. For an axoneme with a fixed CP such as that from sea urchin sperm [44, 45], the twist rigidity of the axoneme is presumably high and thus could result in a planar flagellar shape. For the axoneme with a rotating CP such as *C. reinhardtii* flagella, the twist rigidity is probably low, which results in a larger 3D component of the flagellar shape. It would be interesting to study whether the CP of human sperm also rotates. Finally, it has been suggested that the CP is necessary to generate a planar beat pattern because motile cilia and flagella lacking CP (axonemal structures 9$+$0) exhibit pronounced non-planar beat patterns [46–49].

## Methods

### Sample preparation

Sea urchin (*Arbacia punctulata*) sperm samples were provided by the Marine Biological Laboratory in Woods Hole (Massachusetts, USA). Dry sperm were studied at $1:10^{{6}}$ dilutions in artificial seawater (ASW) containing 0.5% Pluronic [50]. The temperature was maintained at $18^{\circ }\hbox {C}$ by an incubation box (Life Imaging Service, Switzerland). Human sperm were collected by masturbation of healthy donors. A “swim-up” procedure was used for purification. The purified human sperm were studied at $37^{\circ }\hbox {C}$ in the human tubal fluid (HTF) solution containing 3 mg/mL human serum albumin (HSA).

### Optical setup

DIHM was performed in an inverted microscope (IX 71; Olympus) with an oil-immersion objective (40x, UPlanFL N, NA 1.3; Olympus) together with 1.6x magnifying lens in the microscope. A laser (510 nm, LDH-D-C serials, PicoQuant GmbH) driven by the corresponding controller (Sepia II Multichannel Processor, PicoQuant GmbH) was used as a coherent light source. The laser light was coupled into a multi-mode fiber and guided into the microscope. The fiber was held using a custom-made adapter onto the position of the bright light condenser. Sperm cells were placed in a custom-made observation chamber with a depth of $150\,\upmu \hbox {m}$. The hologram was recorded by a fast monochrome camera (pco.dimax; PCO AG) at 1000 frames per second.

### Reconstruction of 3D information from holograms

Rayleigh-Sommerfeld backpropagation was performed for numerically calculating the hologram image at different heights as previously described [51]. This method is valid for weakly scattering objects. To pinpoint the height at which the different parts of the cell come into focus, a height direction gradient filter based on the Gouy phase shift was used [52]. Extracting the 3D coordinates of the object from the refocus stack was done using a filament searching method. This method defines a cone in 3D with the apex fixed at one ending of the filament, then rotates the cone around its apex in yaw ($\pm 45^{\circ }$) and pitch ($\pm 30^{\circ }$) range to find the position of the nearby volume for which the integral of the pixels within the cone is maximal. The center of pixel intensity within the cone at this position is set to be the next control point, and the process is iterated until the centerline of the flagellum is obtained. The three-dimensional tracking data of the sperm flagella shown in Figs. 2, 3, 4 and S1 are available for download at https://github.com/penstand/CurvatureAndTorsion.

### Curvature and torsion computation

Numerical computation of torsion of space curves, which comprise measurement noise and possibly inflection points, poses a trade-off between unavoidable artifacts and robustness. To calculate curvature and torsion of flagellar shapes, we resorted to the original, elementary-geometric definition of curvature and torsion in terms of osculating planes and circles (Fig. S2). Our algorithm is available at: https://de.mathworks.com/matlabcentral/fileexchange/47885-frenet_robust-zip. We used the following parameters: sliding window length, $5\,\upmu \hbox {m}$; regularization weight to enforce continuity of the Frenet-Serret frame, 0.1. Note that the Frenet-Serret formulas for curvature and torsion from differential geometry are mathematically equivalent, but can be difficult to apply to noisy measurement data, because these formulas involve high-order spatial derivatives.

Specifically, in our algorithm for robust computation of curvature and torsion, we used a sliding window moving along the flagellar centerline. For each windowed flagellar segment, we determined the local tangent vector and the local osculating plane (spanned by the local tangent and the local normal vector) by a least-square fit of a straight line and plane to the corresponding region of the flagellar shape, respectively (Fig. S2). A regularization prior constrains the rate of rotation of the osculating plane as function of arc-length. Taubin’s method of fitting a circle [53] was then used to determine the osculating circle within the osculating plane. The unsigned local curvature is the inverse radius of this osculating circle.

Finally, flagellar torsion was computed from the rotation of the local osculating plane along the flagellar centerline, corresponding to the rate of rotation of the local binormal vector around the local tangent vector.

### Sign convention for curvature

We use a convention of a signed curvature that agrees in absolute value with the usual unsigned curvature but can have either positive or negative sign. For a curve in two-dimensional space, a positive curvature corresponds to a counter-clockwise rotation of the tangent vector if the curve is transversed in proximal-to-distal direction (convex bend), while a negative curvature corresponds to a clockwise rotation (concave bend). For a curve in three-dimensional space, we require that the Frenet-Serret frame consisting of tangent vector **t**, normal vector **n**, and binormal vector $\mathbf{b} =\mathbf{t} \times \mathbf{n}$ changes continuously along the curve. Then, a positive curvature corresponds to a positive rate of rotation of the Frenet-Serret frame around the vector **b** when the curve is transversed in the proximal-to-distal direction, while a negative curvature corresponds to a negative rotation rate (see Fig. S3). We note that it is only possible to define a sign of the curvature when the curves have a designated proximal and a distal end, as it is the case for sperm, where we arbitrarily use the flagella end near the head as starting point for the arclength coordinate *s*. For curves in three-dimensional space, the global sign of the entire curvature profile as a function of arclength *s* and time *t* (as shown e.g. in Fig. 4a) is not determined, i.e., $\upkappa (s$,*t*) and –$\upkappa (s,t)$ are equivalent. This gauge freedom results from the fact that for a continuous Frenet-Serret frame **t**(*s*, *t*), **n**(*s*, *t*), **b**(*s*, *t*), there is a second choice of a continuous Frenet-Serret frame given by **t**(*s*, *t*), -**n**(*s*, *t*), -**b**(*s*, *t*). Intuitively, this ambiguity is analogous to a counterclockwise rotation in a 2D plane viewed from above, which becomes a clockwise rotation if the same plane is viewed from below instead.

### Cross-correlation between curvature and torsion waves

We compute the normalized cross-correlation $C(s,\Delta t)$ between curvature and torsion in Fig. 4c (blue curve) as

3 $$\begin{aligned} C(s,\Delta t)=\frac{\left\langle {\vert \upkappa (s,t) \vert \uptau (s,t+\Delta t)} \right\rangle -\left\langle {\vert \upkappa (s,t)\vert } \right\rangle \left\langle {\uptau (s,t)} \right\rangle }{\sigma _{\left| \upkappa \right| } \sigma _{\uptau } }\nonumber \\ \end{aligned}$$

where $\sigma _{{\vert \upkappa \vert }}$ and $\sigma _{{\uptau }}$ denote the standard deviation of $|\upkappa (s,t)|$ and $\uptau (s,t)$, respectively. For Fig. 4c, we plot *C*(*s*, $\Delta t)$ averaged over a small range 29.5–30.5 $\upmu \hbox {m}$ of arc-length positions. Note that the computation of torsion is more reliable in the middle part of the flagellum compared to the proximal or distal part.

The auto-correlation function *A*(*s*, $\Delta t)$ of the curvature (red curve in Fig. 4c) was computed analogously as

4 $$\begin{aligned} A(s,\Delta t)=\frac{\left\langle { \upkappa (s,t) \upkappa (s,t+\Delta t)} \right\rangle -\left\langle {\upkappa (s,t)} \right\rangle ^{2}}{\sigma _{\upkappa }^{2} } \end{aligned}$$

where $\sigma _{\mathrm {\upkappa }}$ denotes the standard deviation of $\upkappa (s,t)$. We appreciate that the auto-correlation function *A*(*s*, $\Delta t)$ displays characteristic (damped) oscillations at the frequency of the flagellar beat, as expected. The cross-correlation function *C*(*s*, $\Delta t)$ between the absolute value of the curvature and the torsion displays oscillations with the same oscillation period but phase-shifted by roughly one-quarter oscillation period. This is consistent with traveling torsion peaks phase-locked with the traveling curvature waves, with torsion peaks positioned near the low-curvature flagellar regions. In particular, torsion waves have the same wavelength and wave speed as curvature waves.

**Note added in proof.** Ref. [25] has been retracted due to potential issues with data analysis.

## Supplementary Information

Below is the link to the electronic supplementary material.

**Supplementary Fig. 1.**\textbf{Gallery of} \textbf{flagellar shape, curvature} ($\kappa $), \textbf{and torsion} ($\tau $) \textbf{of human sperm.} (\textbf{a-e}) Kymographs of the flagellar curvature (left) and torsion (right) for five additional human sperm cells (a-e), analogous to fig. 4(a-b) in the main text. Dark red missing values.

**Supplementary Fig. 2.**\textbf{Computation of curvature and torsion using osculating planes and circles.} (\textbf{a}), Our computation of the Frenet-Serret frame relies on its elementary-geometric definition in terms of an osculating plane (yellow) and osculating circle (dashed red) at each arc-length~position of the flagellar shape (green dot), see methods for details. (\textbf{b}), Flagellar shape (gray) together with computed Frenet-Serret frame consisting of tangent vector (green), normal vector (blue), and binormal vector (red).~~Black arrows represent the laboratory reference frame.

**Supplementary Fig. 3.**\textbf{Signed curvature.} The Frenet-Serret frame defines the tangent vector (green), normal vector (blue), and binormal vector (red circles) at each point of the curve. (\textbf{a}) In mathematics, often unsigned curvature $|\kappa |$ is used, which is always positive. For this definition, the Frenet-Serret frame can change discontinuously at the inflection point of the curve, where the Frenet-Serret frame is not defined. (\textbf{b}) For curves in two-dimensional space, a common convention assigns a positive (negative) sign to the curvature when the tangent vector rotates counterclockwise (clockwise) in the direction along which the curve is transversed - here in a proximal-to-distal direction. For curves in three-dimensional space, we can require that the Frenet-Serret frame changes continuously along the curve. It is then possible to define a signed curvature analogous to the 2D case: the sign of curvature is positive (negative) if the rate of rotation of the Frenet-Serret frame around the binormal vector (vector pointing towards the reader, red circle) is positive (negative). (\textbf{c}) Same as (b), but with binormal vector pointing away from the reader (red points). The normal vector (blue) is now flipped and the sign of the curvature is opposite. Intuitively, this is similar to a rotation in a plane that appears clockwise when viewed from above but becomes a counter-clockwise rotation when the plane is viewed from below. Generally, the global sign of an entire curvature profile as a function of arclength $s$ (and possibly time $t)$ is not determined, i.e., $\kappa (s$,$t)$ and --$\kappa (s$,$t)$ are equivalent.

**Supplementary Movie 1.** Sequence of three-dimensional flagellar shapes from a sea urchin sperm in the stationary laboratory {\it xyz}-frame where the {\it xy}-position of the head center has been aligned. Arrows are $20\,\rmu\myhbox{m}$ long. The flagellar-beat envelope of sea urchin sperm is almost flat. Only one every seven flagella recorded are shown.

**Supplementary Movie 2.** Sequence of three-dimensional flagellar shapes from a human sperm in the stationary laboratory {\it xyz}-frame where the {\it xy}-position of the head center has been aligned. Arrows are $20\,\rmu\myhbox{m}$ long. The flagellar-beat envelope of human sperm features a larger variation in the z-direction. Only one every three flagella recorded are shown.
