# Supplementary figures and images for: Reconstruction of the three-dimensional beat pattern underlying swimming behaviors of sperm
Source: Eur Phys J E Soft Matter. 2021 Jul 1;44(7):87. doi: 10.1140/epje/s10189-021-00076-z (PMC8249298; doi:10.1140/epje/s10189-021-00076-z)

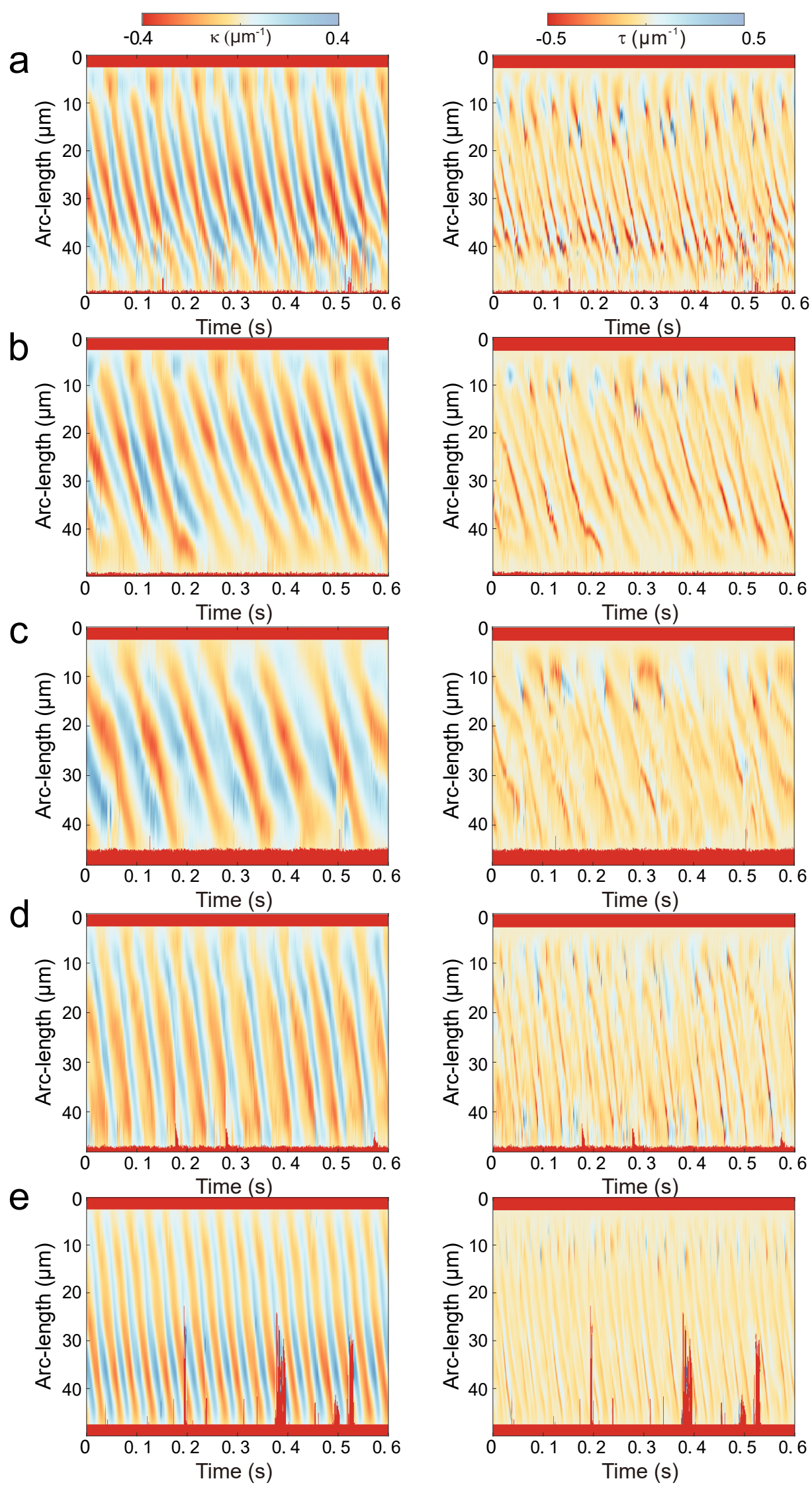

Supplement: Supplementary file 1 — Supplementary Fig. 1.\textbf{Gallery of} \textbf{flagellar shape, curvature} ($\kappa $), \textbf{and torsion} ($\tau $) \textbf{of human sperm.} (\textbf{a-e}) Kymographs of the flagellar curvature (left) and torsion (right) for five additional human sperm cells (a-e), analogous to fig. 4(a-b) in the main text. Dark red missing values. [file 10189_2021_76_MOESM1_ESM.pdf]

a

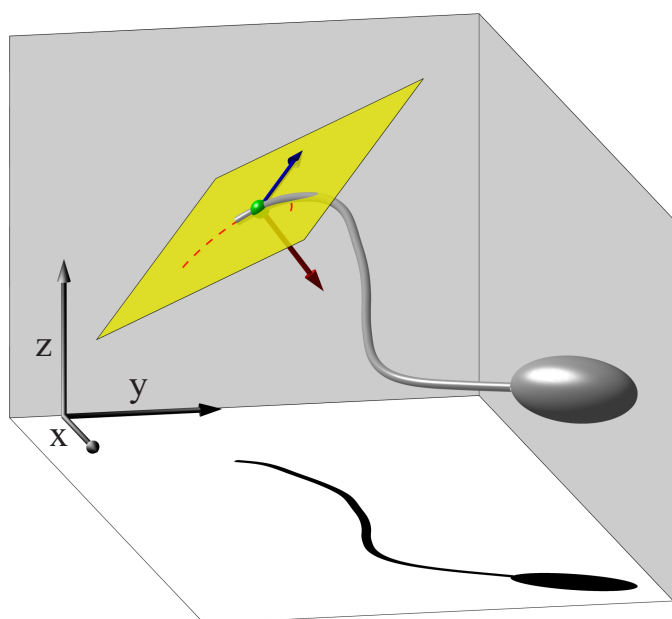

b

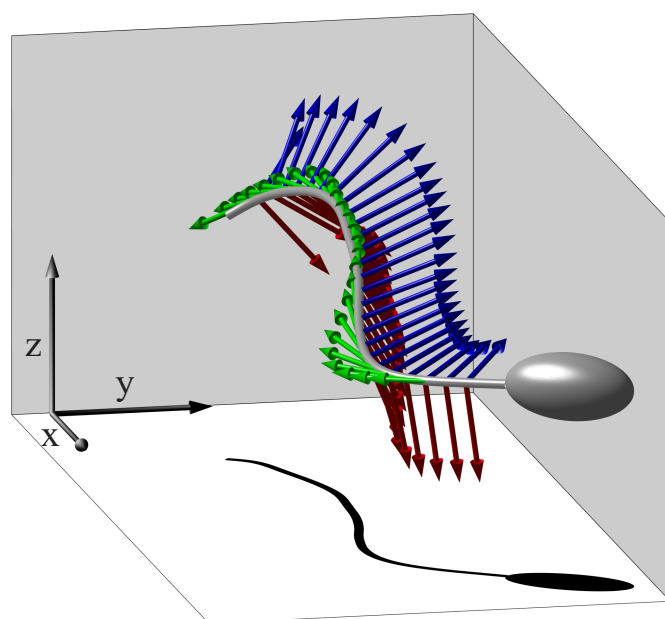

Supplement: Supplementary file 2 — Supplementary Fig. 2.\textbf{Computation of curvature and torsion using osculating planes and circles.} (\textbf{a}), Our computation of the Frenet-Serret frame relies on its elementary-geometric definition in terms of an osculating plane (yellow) and osculating circle (dashed red) at each arc-length~position of the flagellar shape (green dot), see methods for details. (\textbf{b}), Flagellar shape (gray) together with computed Frenet-Serret frame consisting of tangent vector (green), normal vector (blue), and binormal vector (red).~~Black arrows represent the laboratory reference frame. [file 10189_2021_76_MOESM2_ESM.pdf]

a

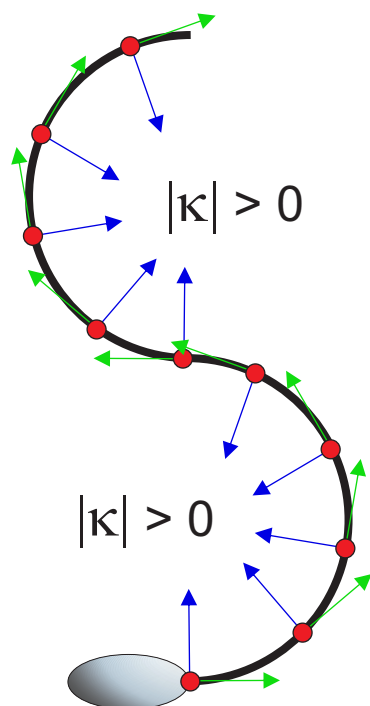

b

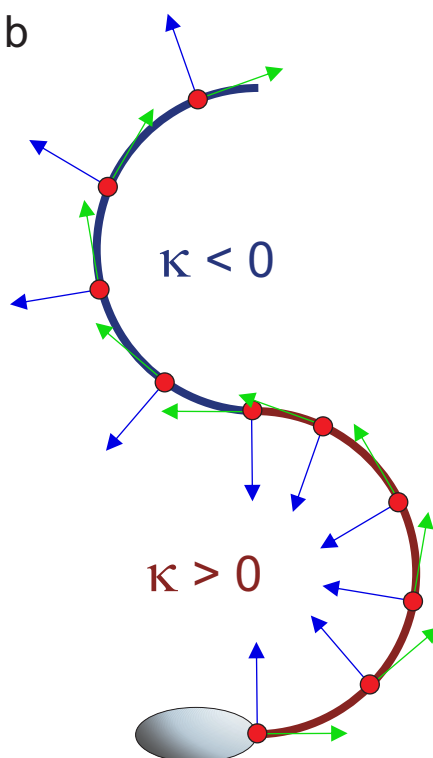

c

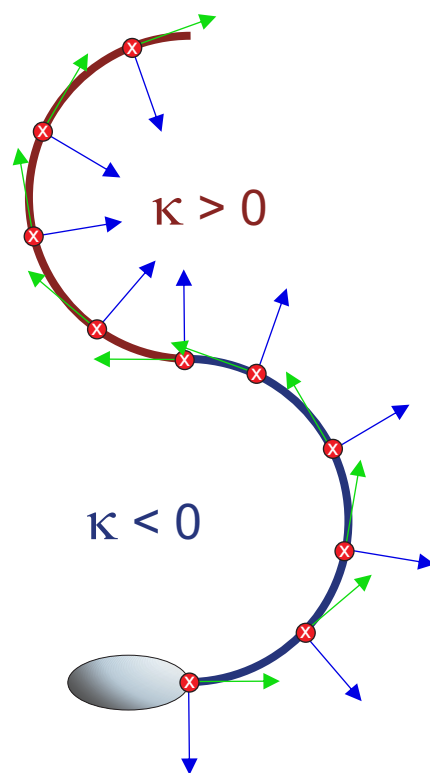

- Pointing towards the reader
- ✕ Pointing away from the reader

Supplement: Supplementary file 3 — Supplementary Fig. 3.\textbf{Signed curvature.} The Frenet-Serret frame defines the tangent vector (green), normal vector (blue), and binormal vector (red circles) at each point of the curve. (\textbf{a}) In mathematics, often unsigned curvature $|\kappa |$ is used, which is always positive. For this definition, the Frenet-Serret frame can change discontinuously at the inflection point of the curve, where the Frenet-Serret frame is not defined. (\textbf{b}) For curves in two-dimensional space, a common convention assigns a positive (negative) sign to the curvature when the tangent vector rotates counterclockwise (clockwise) in the direction along which the curve is transversed - here in a proximal-to-distal direction. For curves in three-dimensional space, we can require that the Frenet-Serret frame changes continuously along the curve. It is then possible to define a signed curvature analogous to the 2D case: the sign of curvature is positive (negative) if the rate of rotation of the Frenet-Serret frame around the binormal vector (vector pointing towards the reader, red circle) is positive (negative). (\textbf{c}) Same as (b), but with binormal vector pointing away from the reader (red points). The normal vector (blue) is now flipped and the sign of the curvature is opposite. Intuitively, this is similar to a rotation in a plane that appears clockwise when viewed from above but becomes a counter-clockwise rotation when the plane is viewed from below. Generally, the global sign of an entire curvature profile as a function of arclength $s$ (and possibly time $t)$ is not determined, i.e., $\kappa (s$,$t)$ and --$\kappa (s$,$t)$ are equivalent. [file 10189_2021_76_MOESM3_ESM.pdf]
